# Toward a Universal Switch for Microbial Granulation: Photogranule Insights to Advance Granular Wastewater Treatment

**DOI:** 10.1021/acs.est.6c05495

**Published:** 2026-10-20

**Authors:** Chul Park, Douglas D. Risser, Yasu S. Morita, Klaus Nüsslein, Joseph G. Gikonyo

**Affiliations:** † Department of Civil and Environmental Engineering, 14707 University of Massachusetts, Amherst, Massachusetts 01003, United States; ‡ Department of Biology, University of Colorado, Colorado Springs, Colorado 80918, United States; § Department of Microbiology, University of Massachusetts, Amherst, Massachusetts 01003, United States

**Keywords:** microbial granulation, Goldilocks zone, c-di-GMP signaling, biofilms, bioengineering

## Abstract

Microbial granulation—the assembly of self-immobilized, spheroidal aggregates—is a transformative approach for high-density, energy-efficient wastewater treatment. By concentrating biomass and enhancing resilience to influent fluctuations, granular systems significantly reduce infrastructure footprints. Current granular technologies primarily leverage upflow continuous reactors or sequencing-batch reactors, where specific external selection pressures are imposed to drive granulation. However, granulation remains neither spontaneous nor reliably predictable, and its fundamental biological drivers are poorly understood. Recent advances in photogranules challenge selection-pressure models. Photogranules form from activated-sludge inocula in hydrostatic and hydrodynamic batches and even in CSTRs where classical selection conditions are weak or absent. Moreover, the recurring emergence of compact spheroidal granules across diverse natural and laboratory systems suggests shared, intrinsic biological drivers rather than purely engineered causes. In this Perspective, we propose that the bacterial second messenger c-di-GMP functions as a conserved, Goldilocks-like molecular switch that integrates diverse environmental cues into a coordinated granulation program. We synthesize transcriptomic, biochemical, and genetic evidence and advance three hypotheses linking c-di-GMP signaling and community physiology to granulation. If validated, targeted modulation of c-di-GMP—by genetic or chemical means—could enable predictable, resilient granulation across a wider range of systems, including the continuous horizontal-flow basins that dominate wastewater infrastructure.

## Graphical abstract

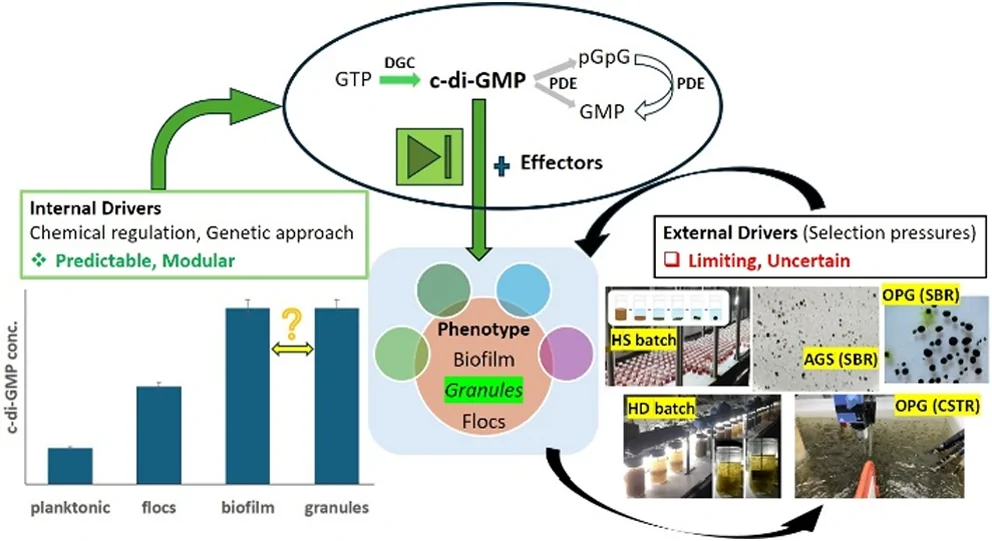

## Introduction

Two decades ago, advanced wastewater treatment was capital- and energy-intensive, requiring complex infrastructure, energy-intensive aeration, and precise control systems. Today, this remains the case and is exacerbated by emerging contaminants such as PFAS and other anthropogenic organics. These demands hinder technology deployment—especially where reliable treatment is most urgent. Further, many Water Resource Recovery Facilities (WRRFs) such as those built after the 1972 Clean Water Act in the US are now reaching the end of their design life. In the US, recent EPA surveys (2024) estimate capital needs to rehabilitate aging WRRF infrastructure at approximately $346 billion over the next two decades.^1,2^ Globally, the capital shortfall is in trillions of dollars.

Microbial granulation offers a promising way to address some of these challenges. Self-immobilized spheroidal microbial aggregates—granules—enable process densification and reduce energy and operating costs,^3^ shrink infrastructure footprints, and improve treatment performance.^4–6^ Their multilayered multifunctional consortia also increase resilience to influent variability.^7^ Notably, oxygenic photogranules (OPGs), dominated by filamentous cyanobacteria, can substitute for mechanical aeration, the single largest energy consumer in WRRFs, by producing oxygen in situ via photosynthesis while sequestering carbon from wastewater and the atmosphere.^8^

However, widescale implementation of granular technologies remains limited by several barriers: dependence on upflow sequencing-batch reactors (SBRs) or upflow-anaerobic-sludge-blanket reactors, slow and inconsistent granulation, risk of granule collapse, and limited policy support.^7,9,10^ For photogranules, providing effective illumination at engineering scale adds an additional challenge.^7^ Granulation in engineered systems is typically driven by selection pressures applied in SBR biotopes—feast/famine cycling, hydraulic selection, and shear—but it is neither always spontaneous nor universally reliable. Granular reactors commonly yield mixtures of suspended cells, flocs, filamentous aggregates, and granules.^7,11,12^ Our limited mechanistic understanding of granulation therefore constrains the transformative potential of granular technologies.

Indeed, granulation is not confined to engineered systems. Analogous spheroidal aggregates appear across nature—in cryoconites on glaciers,^13^ Marimo in lakes,^14^ and many other settings—and they converge on a similar compact spheroidal architecture, regardless of microbial composition, redox regime, or formation pathways (Figure 1).^15–18^ This morphological convergence suggests common internal drivers. Yet the cellular decision to adopt a granular phenotype remains poorly understood. *What genetic or molecular factors trigger this transition? Are they conserved across microbial communities? Can they be engineered?*

**Figure 1:**
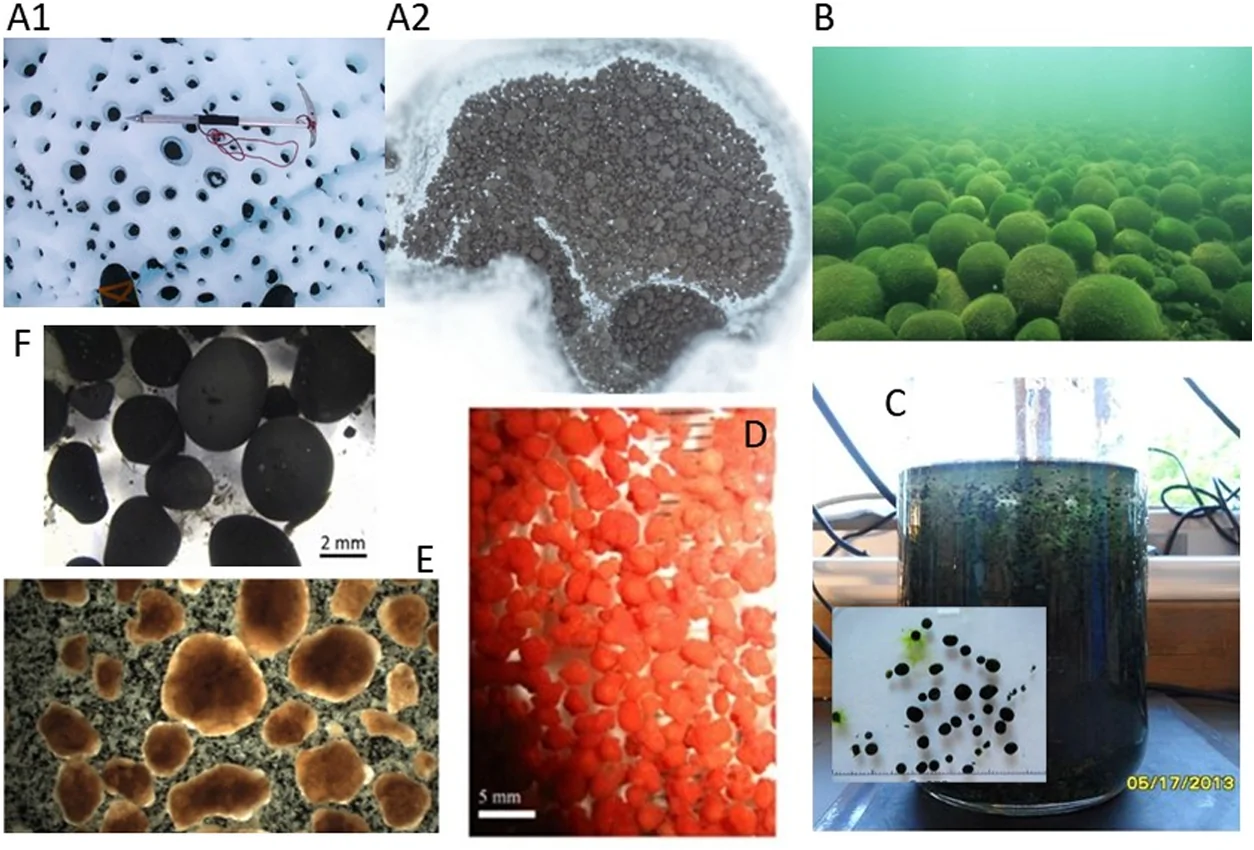
Microbial granules in natural and built environments. (A). Cryoconite granules.^13^ (A1). Melt holes on a glacier surface on the Greenland Ice Sheet. (A2). Cryoconite granules at the bottom of a melt hole. (B). Marimo balls in Lake Akan in Japan.^14^ (C). Oxygenic photogranules. (D). Anammox granules.^17^ (E). Aerobic granular sludge.^16^ (F). Anaerobic granular sludge.^18^ Panels A1, A2, B, E, and F are adapted from references indicated in the caption above, used under a Creative Commons CC BY 4.0 license. Panel D is adapted from Tang et al.^17^ with permission from Elsevier (Copyright 2013 Elsevier B.V.).

Here, we propose that cyclic di-GMP (c-di-GMP), a ubiquitous bacterial second messenger, functions as a conserved molecular switch for granulation. Long recognized for its role in biofilm formation, motility, and extracellular polymeric substances (EPS) production, and recently implicated in specific granulation systems, c-di-GMP integrates diverse environmental cues into coordinated cellular responses. We synthesize evidence across photogranules, anammox granules, aerobic granular sludge (AGS), and aggregates of diverse axenic cultures to propose a unifying molecular framework. Transcriptomic, biochemical, and genetic evidence in this perspective suggests that granulation arises not from a singular reactor type, environmental condition, or selection pressure. It ensues from a “just-right” combination of internal signaling conditions, the Goldilocks Zone,^13,19^ applied at the molecular scale. If correct, this framework suggests a practical route forward: targeted modulation of c-di-GMP signaling could enable predictable and stable granulation across a wider range of engineered systems, including those outside the operational envelope of conventional granulation technologies.

### Photogranules Reveal a Goldilocks Mechanism for Microbial Granulation

The Goldilocks Zone proposal emerged from experimental observations that progressively expanded the known conditions under which photogranules are generated.^19^ After the initial discovery that activated-sludge inocula unexpectedly formed an OPG in a hydrostatic 20 mL vial,^20,21^ a new hypothesis was posed: OPGs should also be produced from activated sludge in the presence of hydrodynamic shear.^19^ The rationale was simple: many types of engineered granules arise under shear, so OPGs should too—despite earlier observations that mixing prevented OPG formation in those small vials. To test this, hydrodynamic batch experiments were conducted in 1 L glass jars using a 3 × 3 × 3 factorial matrix of conditions—three inoculum concentrations, three mixing speeds, and three light intensities.^19^

The results of this study are shown in Figure 2. OPGs can be generated from activated sludge in batch experiments with mixing. Hydrodynamic conditions produced many small granules rather than a single granule, consistent with the development in a shear field.^19,22^ These granular products were nonetheless homologous to hydrostatically formed OPGs, sharing key structural features, including an Oscillatoriales (filamentous cyanobacteria)-dominated architecture.^19^ Importantly, several batch conditions produced OPGs but not all. Because the parameter space can be varied indefinitely, these findings imply that photogranulation is a multifactorial response to environmental stimuli and not deterministically coupled to any single condition.

**Figure 2:**
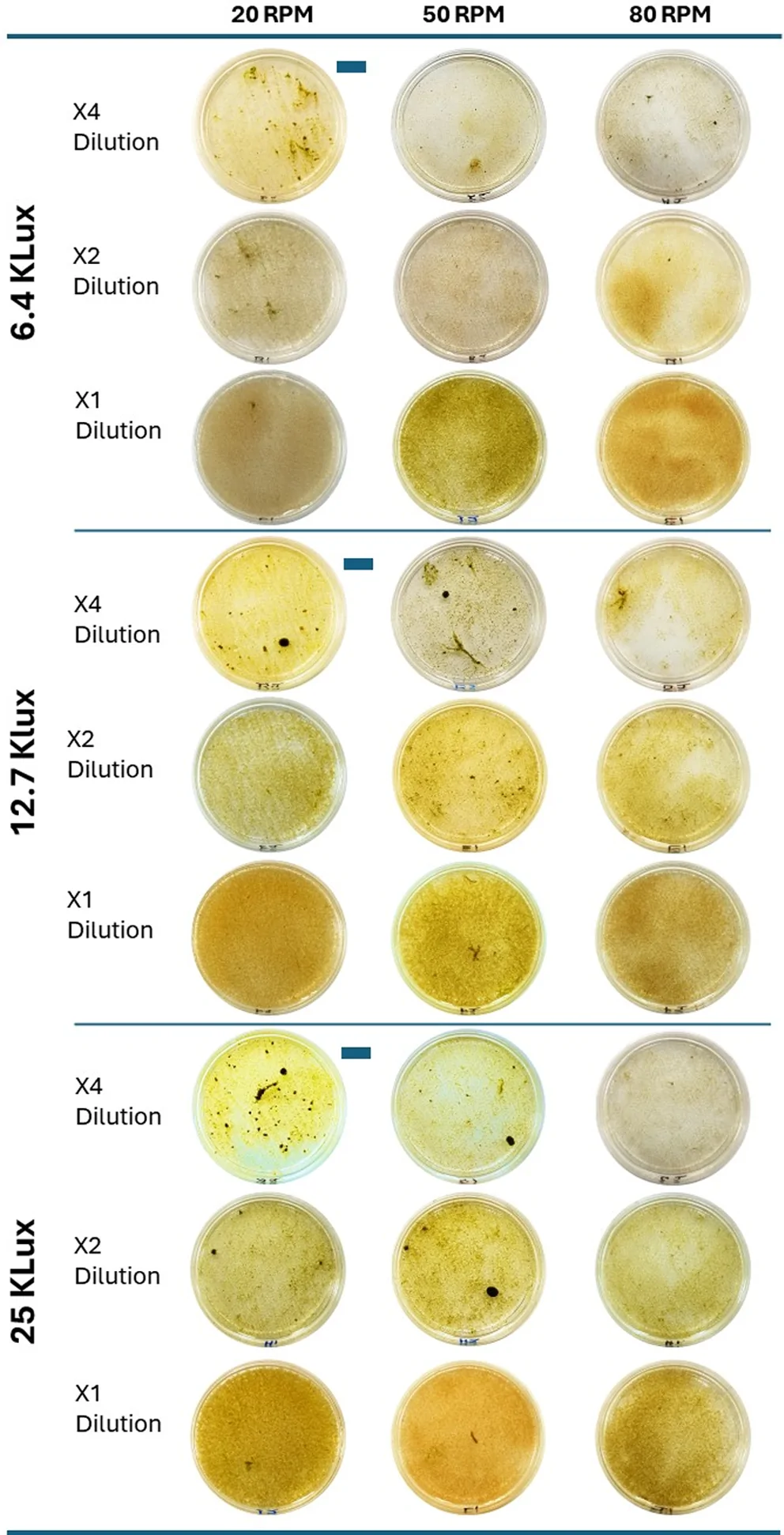
Outcomes of hydrodynamic batch experiments in which activated-sludge inocula were exposed to a 3 × 3 × 3 matrix of conditions.^19^ Different combinations of external (selection) pressures produced multiple OPG granulation zones, while many incubation regimes yielded no readily observable OPG formation. Scale bar: 1 cm. Figure is adapted from Gikonyo et al.^19^ with permission from the Royal Society of Chemistry (copyright 2021 Royal Society of Chemistry).

Today, we know that photogranulation occurs in a wide variety of settings. OPGs are formed from different variations of inocula in hydrostatic batches—lacking shear, feast/famine cycling, and hydraulic selection.^20,21^ As shown above, OPGs can be generated from activated sludge in hydrodynamic batches that include shear but lack feast/famine cycling and hydraulic selection.^19^ Not only in conventional SBRs,^8^ photogranules form in continuous-flow CSTRs treating wastewater without aeration, challenging the long-held view that CSTRs are incompatible with granulation.^7^ And photogranules form even on glacier surfaces—environments that share little beyond the occurrence of granules.^13^ These observations show that granulation is not tied to any specific environmental regime; rather, granules occur whenever the conditions are “just right”.^13,19^ The mechanism of this system-level response at the cellular scale is addressed below.

### Photogranulation of a Single-Species Cyanobacterium Suggests a Signaling Process Involved in Granulation

Formation of ball-shaped aggregates by monoclonal Oscillatoriales was reported as early as the 1960s. In the study of Castenholz,^23^ a single dense ball formed within 30 min from a high-density cell suspension in a still liquid medium, driven by vigorous gliding motility in response to light. These rapidly formed aggregates were easily redispersed by shaking, indicating structurally immature granulation—although it remains unknown how these balls would evolve under extended hydrostatic cultivation. Concurrently, a mutant lacking motility showed no ability to aggregate, demonstrating the importance of motility in this initial ball-forming process. More recent work has shown that Oscillatoriales also produce robust, long-lived granules over several days in hydrostatic batch systems commonly used for OPG cultivation.^24,25^ Together, these observations indicate that a single species of cyanobacteria from subsection III (i.e., Oscillatoriales) can generate granular aggregates across a wide range of temporal scales and mechanical stabilities.

Members of Nostocales are also occasionally abundant in OPGs and cryoconites.^13^ These subsection IV cyanobacteria differ markedly from Oscillatoriales in life history and aggregation behavior.^26^ *Nostoc punctiforme*, a well-studied plant-symbiotic species, undergoes multiple differentiation stages. *Nostoc* forms heterocysts for N_2_ fixation and produces short, motile filaments called hormogonia.^27^ After hormogonia glide to and infect host plant tissue, the plant releases hormogonium-repressing factors (e.g., certain carbohydrates) that promote the transition to nonmotile vegetative filaments and establishment of the nitrogen-fixing symbiotic state.^28^

Risser^26^ reported a remarkable photogranulation phenomenon of *N. punctiforme* in which cells form a compact spherical granule from a dispersed culture (Figure 3A). In the presence of sucralose—a nonmetabolizable sucrose analogue that mimics carbohydrates supplied by symbiont plants—*N. punctiforme* grew primarily as nonmotile vegetative filaments and remained dispersed in water. However, upon removal of sucralose and resuspension in media, cells strongly self-aggregated and formed a photogranule within 24 h. Microscopy revealed that after the removal of sucralose, *Nostoc* exhibited synchronous hormogonia development. This substantial degree of the restored motility—with the attendant increase in cell–cell collisions and contacts—seemed to drive the *Nostoc* photogranulation.^26^ Consistent with a motility-dependent mechanism, a nonmotile Δ*pilB* mutant did not granulate by the same treatment (Figure 3B).^26^

**Figure 3:**
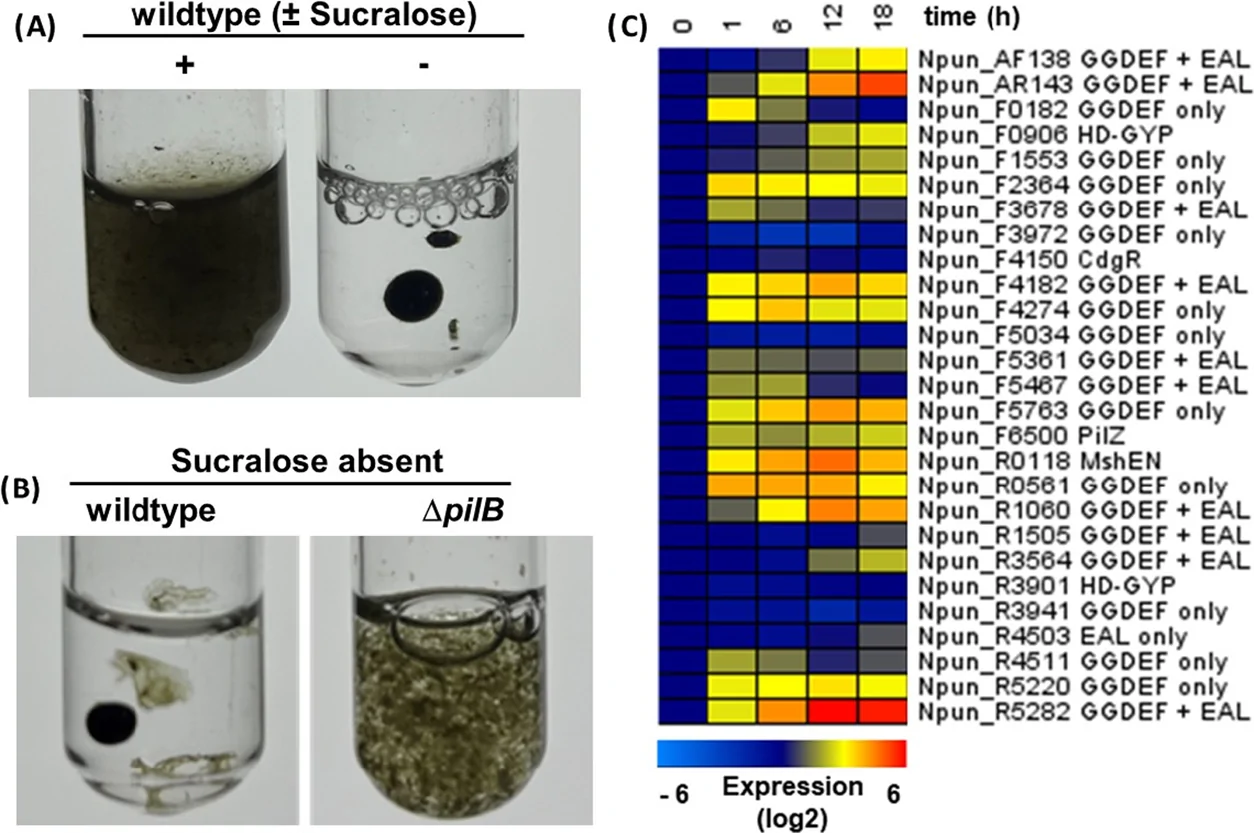
Photogranulation of *Nostoc punctiforme*. (A) Wild-type *N. punctiforme* grows dispersed in the presence of sucralose. Once sucralose is removed and cells are resuspended in growth media, they strongly granulate.^26^ (B). The nonmotile Δ*pilB* mutant strain does not granulate by the same treatment, indicating a crucial role that motility plays in this granulation process.^26^ (C). Gene expression of c-di-GMP signaling genes during photogranulation in *N. punctiforme*. Expression normalized to *t* = 0 h. Panels (A) and (B) are adapted from Risser (2025),^26^ used under a Creative Commons CC BY 4.0 license.

Reanalysis of *N. punctiforme* transcriptomic data in Gonzalez et al.^29^ showed that this dispersed-to-granular transition was accompanied by substantial changes in c-di-GMP regulation genes (Figure 3C). *N. punctiforme* harbors 27 genes encoding proteins putatively involved in c-di-GMP signaling.^30^ Three are likely effectors for c-di-GMP, and 24 are probably c-di-GMP metabolizing enzymes based on the presence of diguanylate cyclase (a GGDEF motif) or phosphodiesterase (either HD-GYP or EAL motifs) domains.^30,31^ Transcriptomic profiling during the 18 h process of photogranulation indicates that 20 of these 27 genes are upregulated, including several that are strongly upregulated.

How c-di-GMP regulates motility in *N. punctiforme* is unknown,^26^ and it is unclear whether elements of the c-di-GMP regulatory cascade shown in Figure 3 are the cause or the consequence of synchronous hormogonium development. Nonetheless, it is clear that motility is essential for photogranulation of *N. punctiforme* and c-di-GMP signaling is implicated in that process. This finding may appear paradoxical because, in most bacteria, elevated c-di-GMP typically represses motility and promotes surface-attached biofilm formation.^32,33^ We will further address this in the later sections. Finally, evidence indicates that photogranulation is not restricted to Oscillatoriales. Although Park and Takeuchi emphasized Oscillatoriales in photogranulation,^13^ *Nostoc* form photogranules and recent work—including our hydrodynamic batch work—shows that unicellular *Synechocystis* (subsection I) can also photogranulate.^34,35^

### c-di-GMP and Granulation

The dinucleotide bis(3′ → 5′)-cyclic diguanylate (c-di-GMP) is a ubiquitous bacterial second messenger whose importance has been recognized since its discovery.^36^ C-di-GMP controls many aspects of surface-associated behavior—adhesion, biofilm formation, motility, and production of EPS—all relevant for granular aggregation.^30,31,37^ Intracellular levels are set by opposing enzymatic activities: diguanylate cyclases (DGCs) with a GGDEF domain synthesize c-di-GMP, and phosphodiesterases (PDEs) with EAL or HD-GYP domains degrade it.^30^ These enzymes occur as individual DGCs or PDEs or as composite bifunctional proteins (GGDEF/EAL), and are frequently fused to or linked with sensory/regulatory domains (GAF, PAS/PAC, REC, etc.), enabling direct coupling of environmental perception to c-di-GMP homeostasis.^38,39^

We propose that c-di-GMP functions as a *common molecular switch for microbial granulation*. Central to this idea is the multifactorial architecture of c-di-GMP signaling. Microbes employ intricate c-di-GMP signaling circuits to modulate their behavior in response to a broad repertoire of physicochemical cues. Srivastava and Waters^33^ documented that c-di-GMP networks are responsive to various stressors—light, oxygen, surface contact, nitric oxide, arginine, and quorum-sensing signals, among others. In these circuits, DGCs and PDEs both receive input from upstream or their own sensors/regulators and communicate with downstream c-di-GMP-binding effectors, enabling tight spatial and temporal control of the nucleotide pool.^38^ The integration of multiple context-dependent sets of receptors/effectors^30^ may explain the funneling of different combinations of first messengers (environmental conditions) into the same aggregate-forming outputs: the molecular basis of the Goldilocks zone. Conversely, modest shifts in the balance, timing, or combination of these inputs can move the network away from the “just-right” state and thereby suppress granule formation.

Empirical evidence across systems supports a central role for c-di-GMP in granulation. Guo et al. (2017a)^40^ showed that anammox-enriched cultures exposed to short (12 h) unfavorable conditions—aerobic exposure and low pH—developed larger granules coincident with elevated c-di-GMP and increased EPS production. In SBR-operated anammox sludge, feed-induced nitrogen stress produced faster granulation alongside an increase in c-di-GMP, extracellular proteins, and precursors for specific EPS.^41^ Recent photogranulation studies also report c-di-GMP involvement. Kong et al.^25^ observed up-regulation of a chemosensory-like Wsp system genes and DGC genes in Oscillatoriales associated with enhanced OPG formation. Most recently, Shi et al.^42^ documented increasing c-di-GMP during photogranulation of activated-sludge inocula amended with Oscillatoriales in hydrostatic cultivation. Together, these observations indicate that elevations in c-di-GMP and stimulation of EPS synthesis are recurring features that accompany granulation, despite substantially different microbial and environmental systems in which granulation occurred.

### Why Granular?

A core question remains unresolved. Microbial aggregations—microbial communities encased in EPS—arise in two architecturally related but distinct forms: surface-attached *biofilms* and self-immobilized suspended aggregates including *granules*. Elevated c-di-GMP is classically associated with biofilm formation, yet emerging evidence now implicates the same signal in granulation.^40,41,43^ *Why does the same signal hub produce either surface-attached biofilms or granules?* If spatial form is a continuum as theorized by Aqeel et al.,^11^ how about suspended flocs? This paradox has a practical dimension. In hydrostatic OPG cultivations, identical activated-sludge inocula under nominally identical conditions can yield either spherical granules or an attached mat, with both aggregates dominated by Oscillatoriales.^13,24^ In other granular systems such as AGS, a similar phenotypic array exists.^44^ Similar selection pressures, in the same biota, produce different phenotypes—and we do not know why. Joosten et al.^24^ showed that the morphotype of OPGs is determined not merely by Oscillatoriales’ presence, but by which species wins the biochemical competition under prevailing conditions. We propose that the *biochemical competition* is the cause of granulation; species sorting is its readout. A related anomaly is that elevated c-di-GMP is well-known to repress motility associated with biofilms,^33^ so why is increased motility essential for the granulation of *Nostoc* and Oscillatoriales? Whether c-di-GMP simultaneously promotes synthesis of a specific suite of EPS and permits motility through distinct, pathway-specific signaling modules—rather than acting as a single global switch—similarly remains unresolved.

The framework of Römling et al.^30^ provides a structure for addressing these questions: Does granulation require activation of multiple c-di-GMP targets in combination—e.g., motility regulation, specific EPS chemistries, lectins or other adhesins—or is a single effector sufficient? Are those targets controlled by one signaling pathway or by several acting in concert? The answers likely differ across organisms and reactor conditions, which is itself revealing: it would imply that granulation is not a single conserved output but a convergent phenotype reachable through multiple routes within the c-di-GMP network. We propose three hypotheses that begin to address these questions.

First, a threshold concentration of c-di-GMP may be needed for granulation, one that exceeds that for surface-attached biofilms. It is well-known that higher c-di-GMP is associated with attached biofilms compared to planktonic cultures. Barraud et al.^45^ measured 101 pmol/mg cellular protein in *P. aeruginosa* biofilm versus 30 pmol/mg protein in planktonic cells. Shi et al.^42^ reported the transient intracellular c-di-GMP concentrations during hydrostatic cultivation of OPGs. The estimated peak level after 20 days’ incubation was 431 pmol/mg protein (119 μg/g biomass), a 3-fold increase from its nongranular baseline on day 5. Likewise, anammox granules had a parallel 4-fold increase above seed-biomass baseline: 10 pmol/mg protein → 40 pmol/mg protein.^41^ Despite a 10-fold difference between two taxonomically distinct granulation systems, a relative increase above a system-specific baseline rather than a fixed absolute concentration may be the toggle between planktonic and sessile states. The case for this “amplitude-based phenotype differentiation” is reinforced by recent evidence that swarming motility in *E. coli* can be induced at only specific c-di-GMP levels by surfactant-acting (amphiphilic) polysaccharides, instead of biofilm formation.^46^ The more tractable near-term question may not be “What is the threshold?” but “Does the granule-forming phenotype arise at a consistently higher c-di-GMP level than the biofilm-forming phenotype when both emerge from the same inoculum?” Comparing granules, attached biofilms, flocs, and suspensions from the same batch, as Shi et al.^42^ have partly begun to pursue, is exactly the kind of controlled comparison needed to test this.

Second, granulation may require c-di-GMP activation by a specific suite of DGC/PDE and downstream effectors in the right combination. Emerging literature shows that many DGC/PDE enzymes function in local, pathway-specific signaling modules that modulate nearby effectors without substantially changing the global c-di-GMP pool.^47,48^ Consequently, DGC/PDE ligands such as O_2_ can produce highly localized and time-limited physiological effects^49^ that may tip the system toward or away from granulation, depending on which modules they engage. Supporting this view are genetic mutation studies of DGC/PDE that reveal an effector-specific response for phenotype expression.

Chen et al.^50^ showed that deletion of EAL-domain PDE genes in *Listeria monocytogenes* led to strong repression in cell motility, an effect correlated to the number of PDE genes deleted. Importantly, the all-PDE-deleted mutant strain (Δ*pdeB*/*C*/*D*) strongly self-aggregated and settled in liquid medium within 2 min,^51^ a rapid settling characteristic resembling granular aggregates. Strikingly, this mutant did not develop any significant biofilms on abiotic surfaces (polystyrene, metal, and glass), and EPS produced in this mutant seemed to inhibit its biofilm formation but promoted self-aggregation.^51^

Köseoğlu et al.^51^ identified the EPS produced in the *L. monocytogenes* PDE mutants as N-acetylmannosamine and galactose-based polysaccharides. Deletion of any gene in this EPS biosynthesis machinery operon abolished self-aggregation and cells grew planktonic like the wild type. Similarly, deletion of *dgcA* or *dgcB* but not *dgcC* abrogated the aggregate phenotype. This cascade of polysaccharide arising from PDE deletion with specific DGC dependence seems to favor cell–cell over cell-surface interactions. In a related context, deletion of *dgc2* in a nonmotile strain *Leptolyngbya boryana*, an Oscillatoriales, restored its motility but paradoxically enhanced surface biofilm, with no significant change in intracellular c-di-GMP concentrations—attributed to compensation by the remaining eight GGDEF proteins.^52^ These observations cannot be explained by global signal amplitude alone, consistent with the view that phenotypic output depends on which cascade nodes are engaged rather than how much c-di-GMP is present.

Cai et al. showed that deletion of a PDE gene (PA0285)—also designated *pipA* (phage-inducing PDE A)—in *P. aeruginosa* resulted in strong self-aggregation (>90% of biomass).^53^ In this work, well-characterized adhesin effectors associated with biofilms were ruled out as aggregation drivers. In comparison to the *Listeria* triple mutant, this partial knockout (1 of 5 conserved motifs) partially repressed motility and likely engaged a limited yet undetermined set of effectors. Curiously, c-di-GMP concentration in the Δ*pa0285* mutant was 4.6 times higher than the wild type, while the deleted PDE gene was reported to regulate phage defense. In vitro, wild-type cells self-aggregated at both very low and very high concentrations of virus amendments. Similar to canonical selection pressures, this phage defense-aggregation relation was found to be nonlinear.

In *Zymomonas mobiliz*, Cao et al.^54^ identified the naturally evolved molecular mechanism responsible for their self-aggregation. The self-flocculating strain ZM401 had two spontaneous mutations from wild-type ZM4: (1) a frameshift producing a more active cellulose synthase BcsA and (2) a mutation in the bifunctional GGDEF/EAL protein ZMO1055 which compromised PDE activity. Cellulase-induced dispersal confirmed cellulose as the proximate cohesion driver, but neither mutation alone was sufficient for aggregation, indicating cascade codependency. The resulting aggregates settled rapidly from suspension and visually resembled dense granules, though not described as such by the authors, underscoring similarity of product across different fields. Later, Li et al.^43^ characterized all five c-di-GMP turnover proteins in ZM4 and established that ZMO1055 is the key regulator of cellulose-triggered self-flocculation.

Together, these findings imply specificity in (1) internal c-di-GMP circuitry and (2) environmental cues to trigger appropriate local modules. Only when the right local c-di-GMP modules are engaged is the granulation-competent combination of effectors activated. This is why granulation cannot be reliably induced by any single environmental level and why the Goldilocks zone reflects a combinatory requirement at the molecular level. A practical corollary is that engineering granulation by targeting individual DGC or PDE genes may be insufficient if the wrong module is engaged; the intervention must activate the granulation-competent combination of effectors.

The hypotheses above still do not resolve why the same signal c-di-GMP produces granules or flocs in some contexts and biofilms in others. A third hypothesis addressing this phenotype routing posits that the outcome may not solely depend on the c-di-GMP signaling per se, but on the relative availability of suitable substrata. For example, where colonizable surfaces are limiting or absent—as in CSTRs and open water—high c-di-GMP may drive cell–cell adhesion and self-aggregation. Where surfaces are abundant, the same signal may preferentially drive attachment. This would explain *OPGs* and *mat* products from identical activated-sludge inocula under nominally identical hydrostatic conditions: minor differences in early surface contact in individual replicate vials may determine which pathway the c-di-GMP signal is routed through. It is worth noting that a mechanistic inversion is also plausible: surface contact itself triggers c-di-GMP elevation via dedicated surface-sensing systems such as the Wsp and Pil-Chp pathways in *P. aeruginosa*.^55,56^ Surface availability may therefore be upstream of the signal rather than a router of it. Under this inversion, suspended aggregations would arise when cells lack surface contact, activating a qualitatively different c-di-GMP signal profile.^55^ Distinguishing these two causal directions is itself an important experimental question. Testing this hypothesis is experimentally tractable: identical cultures with and without abiotic surface area could be compared for granule yield, attached biomass, EPS composition, and c-di-GMP dynamics in a single factorial experiment.

These postulates must similarly resolve the distinction between suspended matrices: flocs and granules. The amplitude hypothesis (hypothesis 1) may provide partial resolution: if granulation requires a higher c-di-GMP threshold than flocculation, surface routing may determine attachment versus suspension, while amplitude determines granule versus floc within the suspended fraction. These hypotheses are therefore complementary rather than competing. The peak c-di-GMP concentration (119 μg/g biomass) reported for OPG by Shi et al.^42^ matched that of the activated-sludge floc inoculum on day 0. Together, the literature and the *Nostoc* transcriptomic data presented above indicate that c-di-GMP regulation is central to bacterial granulation. The mechanistic details remain unresolved. These initial hypotheses advanced here—on signal amplitude, cascade specificity, and surface routing—are intended to guide experimental interrogation.

### Manipulate c-di-GMP Signaling to Enable and Stabilize Granulation

Full-scale adoption of AGS for mainstream wastewater treatment attests to the fact that selection-pressure strategies for granulation can work. Selection-pressure approaches exploit ecological and mass-transfer mechanisms to favor slow-growing, matrix-producing taxa.^44,57,58^ Nevertheless, the narrow operational window required by SBRs creates a structural barrier for most WRRFs, which are typically designed and operated as horizontally continuous-flow systems. Even where SBRs are feasible, SBR operation does not guarantee persistent granulation: changes in temperature, organic or nutrient loading, hydraulic regime, or pH can move the system outside the set of conditions that sustain granules.

Granulation nevertheless occurs in settings that defy conventional selection pressure wisdom among phototrophs, chemolithotrophs, heterotrophs, and complex consortia such as activated sludge under appropriate conditions. This indicates that granulation is fundamentally biological: it reflects complex cellular physiology and signaling in response to environmental cues. From Goldilocks perspective, granulation occupies a broad source yet limited “just-right” region of environmental space; fluctuations that shift the balance of cues can readily degrade or remove granulation. Environmental pressures within the space may offset one another through compensatory shifts in their magnitude.^19^ The molecular hub of that signaling, as we presented above, is c-di-GMP—and it is therefore c-di-GMP that presents the most direct target for engineering granulation beyond the constraints of selection-pressure approaches.

C-di-GMP signaling is well suited to exert phenotypic control. By modulating activity through small-molecule effectors, cells can rapidly toggle behaviors (e.g., EPS synthesis, motility, adhesion) without the energetic cost and delay of repeatedly synthesizing or degrading large macromolecular assemblies (e.g., cellulose synthase).^49^ The widespread conservation of c-di-GMP systems across bacteria also helps explain why taxonomically and ecologically disparate organisms can produce similar granular phenotypes (Figure 1) when exposed to appropriate combinations of cues. Manipulation of c-di-GMP is already widespread—especially in the biomedical field where the goal is prevention or dispersal of unwanted biofilms. Small, drug-like molecules that inhibit DGCs, enhance PDEs, or otherwise block c-di-GMP signaling have been actively developed.^59,60^

In wastewater engineering, the objective is the opposite: elevating or stabilizing c-di-GMP to promote beneficial aggregation. Wang et al.^61^ reported that exogenous polyamines enhance *Agrobacterium tumefaciens* biofilms. Building on this study, Lin et al.^62^ observed that adding exogenous spermidine to activated-sludge batch reactors with woodchip media enhanced low-temperature biofilm development relative to the control. This enhancement occurred concurrently with increased c-di-GMP levels, elevated EPS production, and stronger biofilm adhesion. These findings suggest that chemical modulation of c-di-GMP is inherently feasible for wastewater systems. The value of c-di-GMP manipulation is not that it provides yet another way to navigate the Goldilocks zone—selection-pressure approaches already do that. Its value is that it expands the zone. By biasing the internal c-di-GMP state toward the granulation-competent combination of effectors, the biological threshold for granulation is lowered, making it achievable and sustainable across a wider range of external conditions. A CSTR lacking feast/famine cycling could sustain granulation,^7^ if the internal signal is appropriately primed. Similarly, granule loss under selection-pressure fluctuations becomes less likely not because the operating window has been widened, but because the system is less sensitive to drifting outside it.

C-di-GMP manipulation buys phenotype headroom, but only when intervention aligns the conditions suggested above; either c-di-GMP rises to a sufficient level, or the right combination of DGC/PDE and effector modules is engaged, and that profile is routed toward suspended aggregation rather than surface attachment. Modulating PDE/DGC enzymes directly addresses the first condition—raising and sustaining the signal. The second condition depends on which local signaling modules are active, shaped by the environmental and operational context of the reactor. The third is the most challenging, but has a tract already embedded in current wastewater treatment practice. Biofilm infrastructure and granular infrastructure are opposite routing decisions potentially made on the same molecular switch. Engineers have been exploiting that switch through surface geometry since the first trickling filter—the molecular framework now makes it possible to exploit it directly. Surface-attached biofilm formation is deliberately engineered through carrier media, fixed-film reactors, and IFAS configurations to maximize surface availability and exploit c-di-GMP-mediated attachment.^63,64^ In those systems, the routing decision—granule vs biofilm—has already been made at the design stage, in favor of biofilm, without the molecular framework to describe it as such.

The c-di-GMP modulation strategy proposed here represents the inverse of biofilm infrastructure: an attempt to route the same molecular switch toward granulation, across a broader scope of engineered systems. The framework offers a biological rationale why this may work: if elevated c-di-GMP consistently favors organisms whose effector repertoire produces granulation—as the cross-system evidence above suggests—c-di-GMP modulation may shift competitive community dynamics without requiring precise compositional control. This remains a hypothesis requiring testing in bench-scale mixed-community reactors as the necessary next step.

Likewise, genetic studies detailed previously provide mechanistic proof-of-concept for both back-end and front-end c-di-GMP manipulation strategies. Translating that proof-of-concept into wastewater practice does not mean engineering communities genetically. Continuous microbiome influx, regulatory constraints, and ecological unpredictability make stable genetic modification of mixed communities rather impractical. Chemical amendment is the deployable analogue: transient delivery of PDE/DGC-modulating compounds can achieve the same internal signal shift that genetic manipulation produces in the laboratory. The challenges for chemical approaches include target specificity, effective delivery and dosing in complex matrices, residues in effluent and biosolids, associated biosafety and regulatory acceptance, and cost competitiveness, all of which need to be assessed before field deployment. Additionally, the potential risks of unintended physiological impacts, such as hyper-stimulated motility or excessive EPS production altering aggregation and function, require investigation. A pragmatic near-term strategy would be combining targeted c-di-GMP modulation with established, controllable environmental practices—e.g., controlled shear, preanoxic selectors, or hydraulic selection, known to promote aggregation. Both the biochemical and operational interventions operate through the multifactorial c-di-GMP response.

*Mycobacterium smegmatis*, encoding a single c-di-GMP turnover gene (*dcpA*, Locus 2196, GGDEF/EAL), offers a tractable entry point for dissecting the mechanistic basis of both strategies before moving to more complex communities. In our recent *M. smegmatis* experiments,^65^ the *dcpA* or *dcpA*ΔEAL overexpression mutants produced more robust and persistent granules than the wild-type (Figure 4). These results provide additional and strong evidence that c-di-GMP modulation can cause granulation. Empirical nuance also matters. Both overexpression mutant strains produced strong granules in mixed liquid culture, but formed pellicles when mixing was absent, showing that some degree of hydrodynamic forcing is still required depending on the organism and its motility.^65^ By contrast, phototrophic systems such as hydrostatic OPG cultivation and *Nostoc* can granulate without mixing, likely because significant gliding motility and filamentous morphology drive cell–cell encounters and consolidation. These examples underscore that c-di-GMP modulation and operational controls are complementary levers, with modulation lowering—but not always eliminating—the need for specific external selection pressures. Optimal intervention will depend on taxon, EPS chemistry, and reactor hydrodynamics.

**Figure 4:**
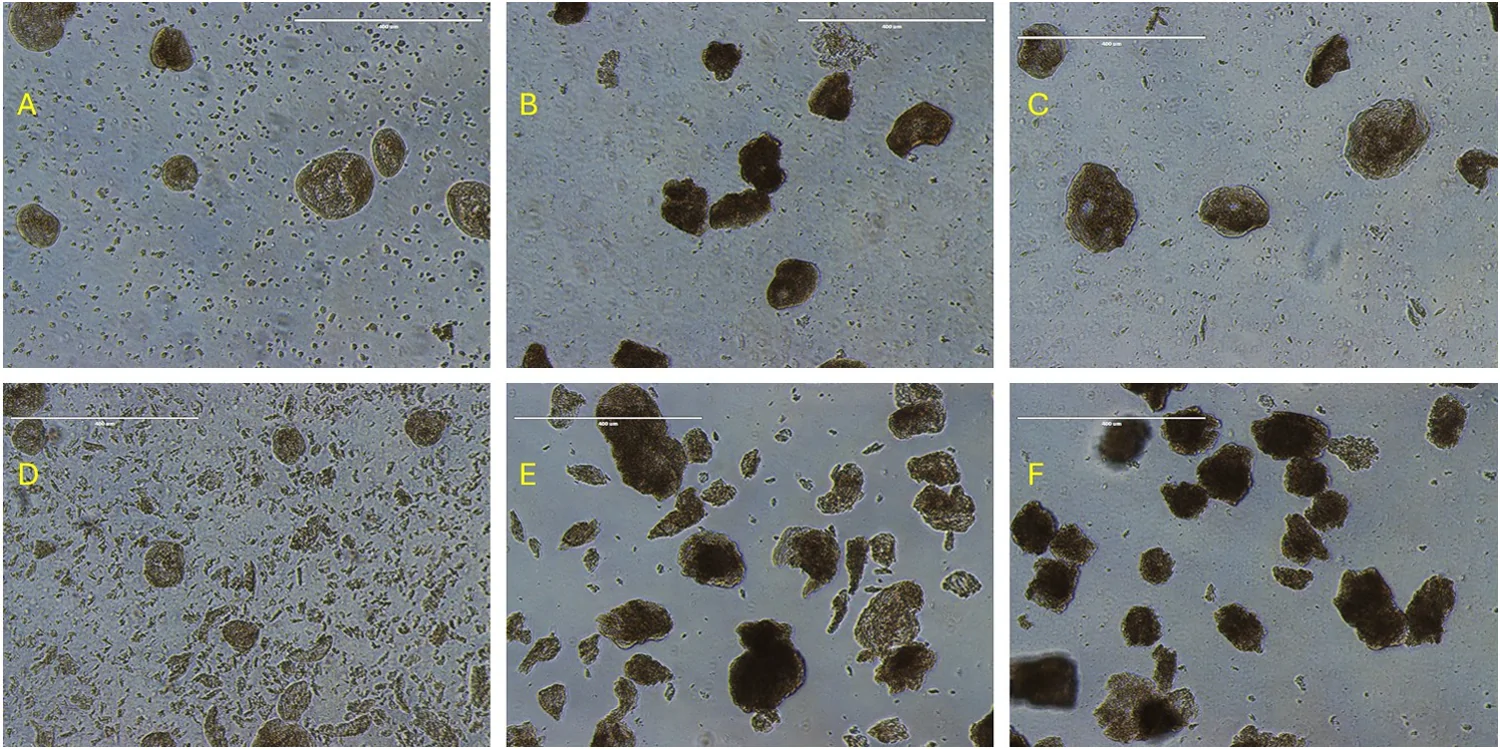
Cellular aggregation phenotypes of wild-type *Mycobacterium smegmatis* and two *dcpA* (GGDEF/EAL) mutant strains following 48 h of growth in standard 7H9 media.^65^ Upper rows (A–C): growth in the absence of a detergent Tween-80. (A). Wild-type *M. smegmatis*. (B). *dcpA* overexpression mutant. (C). *dcpA*ΔEAL overexpression mutant. Lower rows (D–-F): growth in the presence of 0.005% Tween-80. (D). Wild-type *M. smegmatis*. (E). *dcpA* overexpression mutant. (F). *dcpA*ΔEAL overexpression mutant. Scale bars for all panels: 400 μm. While wild-type *M. smegmatis* often forms granular aggregates in shaking batch cultures, note the presence of a background suspension (A) and the strong dissociation that occurs in the presence of a weak detergent (D). Figure is adapted from Lam et al.^65^ with permission from John Wiley and Sons (copyright 2026 Wiley Periodicals LLC).

Figure 5 illustrates our proposal to enhance and sustain granular technologies by engineering the internal driver—c-di-GMP signaling—rather than relying primarily on selection pressures. If successful, this approach could allow existing continuous-flow WRRFs to support robust granulation without conversion to SBR operation. The same logic applies to OPG scale-up. Earlier scale efforts (including a demonstrated scale-up to 120 L CSTRs)^7^ exposed vulnerabilities such as light-granulation feedback that can destabilize granulation. Targeted modulation of c-di-GMP—combined with modest operational controls—could derisk scale-up by making granules more resilient to environmental perturbations. Ultimately, integrating internal (molecular) and external (operational) levers may make next-generation granular wastewater technologies faster to establish, more robust to fluctuation, and more widely deployable.

**Figure 5:**
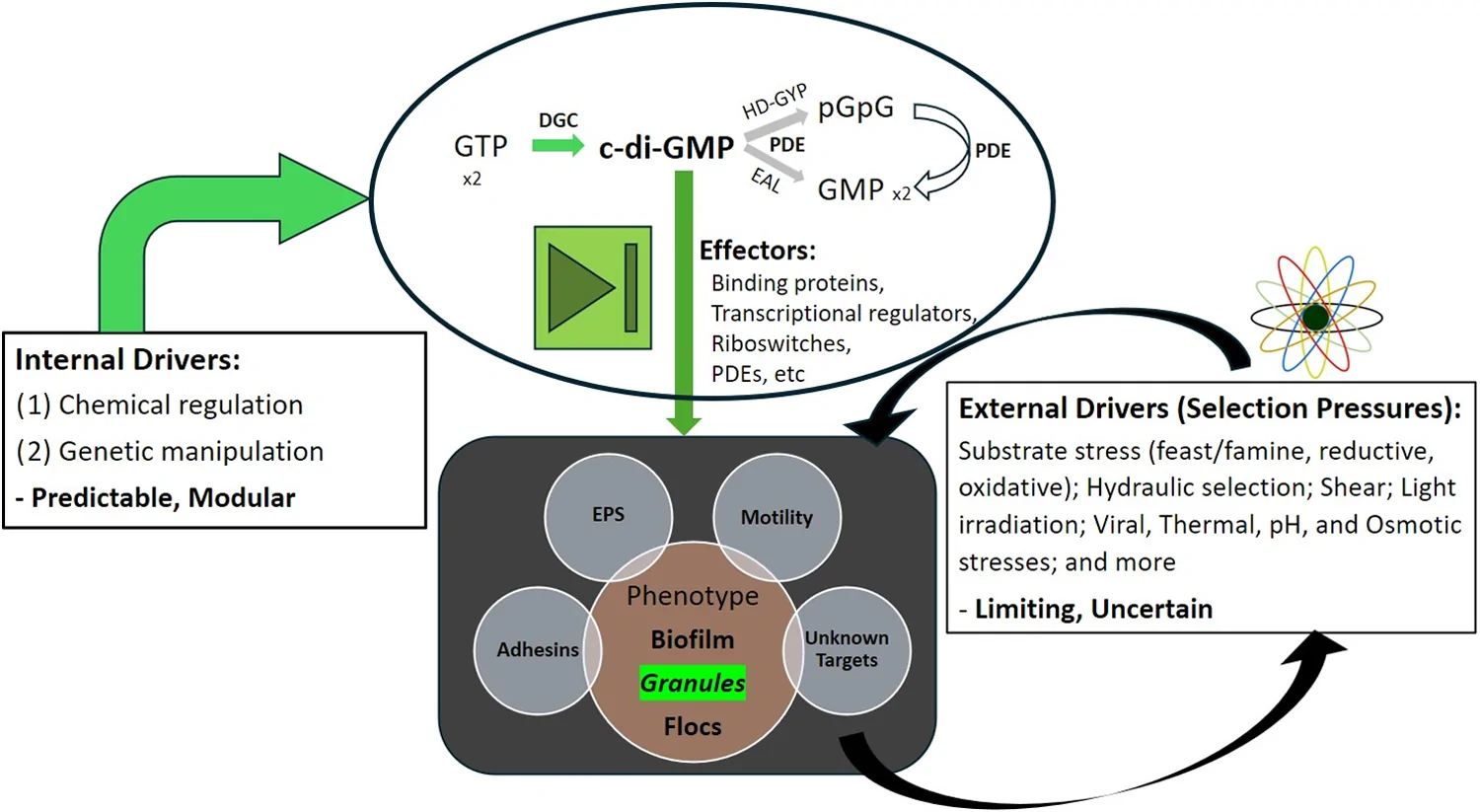
Manipulating c-di-GMP signaling to enhance and stabilize microbial granulation.
